# Teacher Discipline Intensity and Adolescents’ Mental Health Problems: A Moderated Moderation Effect Based on CEPS 2013–2014 Survey Data

**DOI:** 10.3390/bs16071209

**Published:** 2026-07-17

**Authors:** Chunhui Qi, Zhen Zhang

**Affiliations:** Faculty of Education, Henan Normal University, Xinxiang 453007, China; qichunhui@htu.edu.cn

**Keywords:** discipline intensity, violation severity, mental health problems, migration status

## Abstract

Teacher discipline serves as a vital instructional and managerial tool for facilitating adolescent development and exerts profound influences on students’ physical and mental well-being. Grounded in expectancy violation theory, deservingness justice theory, and life history theory, the present study adopts data from the China Education Panel Survey (China Education Panel Survey, CEPS, 2013–2014) to explore the association between teacher disciplinary intensity and adolescents’ mental health problems and rigorously tests violation severity as the first-stage moderator and family migration status as the second-stage moderator within a moderated moderation framework. This study employs cross-sectional data from a nationally representative sample of 17,788 adolescents, and statistical analyses, including descriptive statistics, bivariate correlations, and moderation effect analyses, were conducted using SPSS 24.0 and the PROCESS macro. The empirical results demonstrate significant positive associations between teacher disciplinary intensity, student violation severity, and adolescent mental health difficulties. Family migration status is negatively correlated with perceptions of disciplinary appropriateness and the degree of student violation. Furthermore, violation severity negatively moderates the predictive relationship between discipline intensity and mental health problems. Although intensified teacher discipline significantly predicts higher levels of adolescent mental health problems across both high- and low-violation contexts, such adverse effects are substantially weakened when student violations are severe. Critically, the interactive pattern between discipline intensity and violation severity is more salient for migrant adolescents than for their non-migrant peers. These findings offer a clear theoretical explanation for why reasonable and equitable school discipline can protect and promote adolescents’ mental health.

## 1. Introduction

Mental health refers to a state of being in which an individual has rational cognition, stable emotions, appropriate behavior, harmonious interpersonal relationships, and the ability to adapt to changes during the process of growth and development (Chang et al., 2019). It is an important component of health. Educators have an unshirkable responsibility to cultivate mentally healthy adolescents, and this is also an important task related to social stability and national development. However, with the rapid development of society and the continuous changes in society, the growth environment of children and adolescents is constantly changing, which brings certain challenges to their mental health. Mental health problems are defined as symptoms of mental disorders or self-reported psychological difficulties that cause significant distress and impair daily functioning (Collishaw, 2015). The mental health problems of children and adolescents are increasingly prominent. According to the World Health Organization in 2024, globally, one in every seven adolescents aged 10–19 suffers from a mental disorder, accounting for 15% of the global disease burden in this age group (World Health Organization, 2024). In China, the detection rates of depression, anxiety, sleep problems and self-harm among primary and secondary school students are relatively high, and their overall mental health outcomes are worrying (Yu, 2022). A large number of studies from different disciplines have shown that the mental health status of adolescents not only affects their current physical health and academic performance (Agnafors et al., 2021; Prince et al., 2007), but also profoundly affects their physical and mental health, labor market performance, and social behavior in adulthood (Hale et al., 2015; Koenig et al., 2025; Thomson et al., 2022), ultimately leading to serious consequences such as insufficient overall human capital accumulation and economic losses in society (Black et al., 2022; Stelmach et al., 2022). Therefore, the current academic community urgently needs to explore the potential factors affecting the mental health of adolescents and identify feasible ways to improve their mental health (Piqueras et al., 2024).

Schools constitute students’ core learning environments, and the management practices adopted by schools and teachers exert a profound influence on adolescents’ mental well-being. Teacher discipline has always been a hot issue of concern in families, schools, and society, directly affecting students’ physical and mental health (Qi et al., 2025; Qi & Zhang, 2026; Z. Zhang & Qi, 2024; Z. Zhang et al., 2025). In the context of Western culture, some empirical studies have preliminarily found that the strict discipline implemented by teachers can make students experience negative emotions such as anxiety and depression (Eyllon et al., 2022; Hecker et al., 2016; Valencia et al., 2025). Systematic reviews have also found that exclusionary disciplinary actions, verbal reprimands, and combinations of various disciplinary strategies are all related to students’ mental health problems (Duarte et al., 2023; Ijaz et al., 2024; Wiggers & Paas, 2022). In comparison, although some studies have shown that Chinese parents and students hold positive attitudes towards teacher discipline (Qin et al., 2022; Y. Wang et al., 2021), few studies have explored the impact of teacher discipline on the mental health problems of Chinese adolescents. Given this context, this study systematically investigates the mechanisms through which teacher discipline influences the mental health of Chinese adolescents, thereby contributing empirical evidence to inform educational policy and psychological intervention strategies.

### 1.1. Discipline Intensity and Adolescents’ Mental Health Problems

Educational discipline refers to teachers’ educational behavior of managing, instructing, or correcting students who violate rules based on educational purposes, in order to make students take warning, recognize and correct their mistakes (Z. Zhang et al., 2025). Discipline intensity is defined as the extent of the risk of harm to students stemming from such practices and their corresponding acceptability (Gershoff, 2002), which is generally measured by the severity and frequency of disciplinary actions (Teodorescu et al., 2021; Qi et al., 2025). According to different legal norms in various countries, the intensity range of disciplinary actions ranges from mild verbal criticism to severe expulsion. Although the purpose of educational discipline is to correct problem behaviors and promote students’ development, excessive, overly frequent or malicious disciplinary actions may also affect students’ physical and mental health. Expectation violation theory emphasizes that when others’ actual behavior contradicts one’s prior expectations, people will experience strong discomfort (Burgoon et al., 2016). Therefore, when the intensity of the teacher’s disciplinary action exceeds expectations, students will feel negative emotions such as surprise, confusion and anxiety. Some quantitative empirical studies have confirmed that exclusionary discipline, severe discipline or corporal discipline can lead students to experience stronger symptoms such as depression, anxiety, and behavioral problems (Eyllon et al., 2022; Hecker et al., 2016; Valencia et al., 2025). At the same time, qualitative interview studies have also found that teenagers consider disciplinary management strategies confusing, overly harsh and lacking consistency, which will have negative effects on students’ mental health, well-being and behavioral outcomes (Bell et al., 2026). Moreover, systematic reviews indicate that exclusionary disciplinary practices, verbal reprimands, and combinations of various disciplinary strategies are all significantly associated with poorer mental health and maladaptive behavioral outcomes among students (Duarte et al., 2023; Wiggers & Paas, 2022). Therefore, we propose research Hypothesis 1:

**Hypothesis** **1.** *Discipline intensity positively predicts students’ mental health problems, such that greater disciplinary intensity is associated with more severe mental health problems among students.*

### 1.2. Violation Severity as a Moderator

Violation severity is defined as the evaluative judgment that individuals make regarding the seriousness of a rule infraction (Peterson, 2024). This judgment is grounded in factors such as intent, frequency, and consequences (Eriksson et al., 2017; Z. Zhang & Qi, 2024). When administering discipline to students who have violated institutional rules and regulations, educators must explicitly address the normative basis for sanctioning. Specifically, this basis is the principle of proportionality. This principle, widely upheld in administrative law and disciplinary practice, stipulates that the nature and intensity of a sanction must correspond appropriately to the gravity of the misconduct. Drawing on the theory of just deserts, discipline should be calibrated to reflect the objective seriousness of the violation (Mooijman & Graham, 2018). Appropriately calibrated sanctions not only uphold procedural fairness but also foster constructive teacher–student relationships and support students’ psychological well-being. Conversely, disproportionately severe discipline may engender feelings of alienation, disrespect, and hostility among students, thereby exacerbating internalizing and externalizing mental health difficulties (Chen et al., 2021). Empirical evidence from organizational behavior and school leadership research consistently indicates that fair, transparent, and developmentally appropriate disciplinary practices significantly enhance stakeholders’ perceptions of teacher trustworthiness and trust (Qi & Zhang, 2026; Salcedo & Jimenez-Leal, 2024; L. Wang & Murnighan, 2017). Such practices strengthen collaborative relationships with both students and parents (Qi et al., 2025; Z. Zhang & Qi, 2024; Z. Zhang et al., 2025). Furthermore, a recent large-scale questionnaire study demonstrated that adolescents’ perceptions of unfair or arbitrary school-based discipline serve as a significant positive predictor of subsequent engagement in health-risk behaviors and adverse psychosocial outcomes (Krause et al., 2025). Based on this theoretical and empirical foundation, we propose Hypothesis 2:

**Hypothesis** **2.** *The severity of the violation negatively moderates the association between teacher-imposed disciplinary intensity and students’ mental health problems.*

### 1.3. Migration Status as a Second-Order Moderator

Beyond the potential moderating influence of violation severity, familial migration status represents a pivotal sociodemographic variable that merits systematic investigation. Migrant children, defined as minors whose parents have migrated from rural to urban regions for employment during China’s ongoing socioeconomic transformation (Chang et al., 2019), constitute a unique population shaped by large-scale internal population movement. In this context, children are not merely passive followers of their parents’ geographic relocation; instead, they are integrated into intergenerational mobility trajectories characterized by persistent exposure to structural instability and resource limitations. Life History Theory proposes that developmental trajectories are adjusted in response to key environmental signals, especially environmental predictability and resource accessibility. These cues foster two distinct life history strategies: a fast strategy characterized by accelerated maturation, increased vigilance, and risk-taking decision-making, and a slow strategy characterized by postponed maturation, stronger commitment to long-term goals, and improved self-regulation (Stearns & Rodrigues, 2020; Ellis & Del Giudice, 2019). Accumulating empirical evidence suggests that migrant children endure chronic dual adversity, specifically ecological harshness (e.g., overcrowded living conditions and restricted access to medical care and educational resources) and environmental unpredictability (e.g., frequent residential moves, parental employment instability) (Ellis & Del Giudice, 2019; Han & Chen, 2020). This combined adversity predisposes migrant children to adopt a fast life history strategy. Such an adaptive shift is linked to increased vulnerability to both internalizing problems (e.g., depression, anxiety, and low self-esteem) and externalizing behaviors (e.g., aggressive conduct, rule-breaking, and participation in health-damaging activities) (Han & Chen, 2020). Multiple empirical studies have consistently documented that, compared with their non-migrant peers, migrant children display heightened neural and behavioral reactivity to negative stimuli and suffer from significantly higher rates of depressive and anxiety symptoms (Brance et al., 2024; Chang et al., 2019; Efremov, 2025). Moreover, findings from meta-analyses and systematic reviews strongly confirm these trends, revealing that migrant children consistently bear a heavier overall mental health burden than non-migrant children (James et al., 2022; Salas-Wright et al., 2022; Sun et al., 2015). Drawing on this integrated theoretical and empirical framework, we propose Hypothesis 3:

**Hypothesis** **3.** *Adolescents exposed to residential mobility will demonstrate a significantly stronger moderating effect of discipline intensity and violation severity on mental health problems than their non-migrant counterparts.*

**Figure 1:**
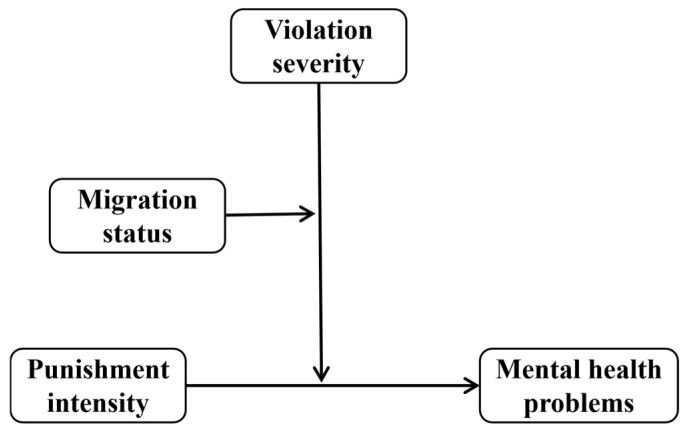
Research model.

In summary, the aim of this study was to explore the association between teacher disciplinary intensity and adolescents’ mental health problems and to rigorously test violation severity as the first-stage moderator and migration status as the second-stage moderator within a moderated moderation framework. The theoretical model for the present study is depicted in Figure 1.

## 2. Materials and Methods

### 2.1. Data Sources

This study employed a secondary cross-sectional analysis using baseline data from a nationally representative longitudinal survey. This study draws on data from the China Education Panel Survey (CEPS), a nationally representative, multilevel longitudinal dataset developed and administered by the China Survey and Data Center at Renmin University of China. CEPS comprises two publicly released waves: the 2013–2014 baseline survey and the 2014–2015 follow-up survey. The China Education Panel Survey (CEPS) set the 2013–2014 academic year as its baseline wave. The survey initially recruited two parallel student cohorts: seventh-grade (Grade 7) and ninth-grade (Grade 9) junior high school students. Stratified random sampling was implemented across China, with the regional average educational attainment of residents and the share of migrant populations serving as stratification criteria. A total of 28 county-level administrative units (counties, districts, and county-level cities) were selected as survey locations. Within these sampled county-level units, schools constituted the primary sampling frame, from which 112 schools and 438 classes were randomly drawn for data collection. A full census of all enrolled students within the sampled classes was undertaken, yielding a baseline sample of roughly 20,000 students.

For the present analysis, we restricted the sample to respondents from the 2013–2014 baseline wave who provided complete responses on all key analytical variables. This yielded an effective analytical sample of 17,788 students. Within this sample, 50.6% were male, 48.2% were in Grade 9, 91.7% identified as Han ethnicity, 54.5% held agricultural household registration (hukou), 17.4% resided in migrant families (defined as households where at least one parent had migrated for work), 32.3% were boarding students, and 44.2% were only children. The mean age was 14.52 ± 1.24 years, and socioeconomic status was generally moderate to high. A total of 1699 participants were excluded, representing 8.7% of the baseline assessment sample. Of these, 612 adolescents had missing data for at least one core demographic variable (e.g., age, sex, or grade level). Among the remaining 1087 participants excluded from the analysis, 40.2% were male; 36.1% were in Grade 9; 86.5% identified as Han ethnicity; 59.5% held agricultural household registration (hukou); 16.5% resided in migrant families (defined as households where at least one parent had migrated for work); 35.1% were boarding students; and 33.8% were only children. The mean age was 13.50 ± 1.21 years, and socioeconomic status was generally moderate to high. Overall, the excluded participants did not differ significantly from those included with respect to key baseline characteristics. Ethical approval for this secondary analysis was obtained from the Ethics Committee of the Faculty of Education, Henan Normal University.

### 2.2. Variable Selection

#### 2.2.1. Predictor Variable

The primary predictor variable in this study is the perceived frequency of teacher-implemented disciplinary actions, as reported by students. Within the Chinese educational and legal context, verbal criticism is a formally sanctioned and widely practiced disciplinary strategy; empirical evidence further supports its efficacy as a mild yet functionally effective intervention in classroom management (Z. Zhang & Qi, 2024). Accordingly, disciplinary intensity is operationalized in this study as the self-reported frequency with which students experience direct disciplinary feedback. This construct is measured using two validated items adapted from the China Education Panel Survey (CEPS) student questionnaire: (1) “My homeroom teacher frequently criticizes me” and (2) “My parents frequently receive critical feedback from my teachers regarding my behavior.” Responses are recorded on a four-point Likert scale ranging from 1 (strongly disagree) to 4 (strongly agree), with higher scores indicating greater perceived disciplinary intensity. The two-item scale demonstrates acceptable internal consistency (Cronbach’s α = 0.63).

#### 2.2.2. Outcome Variable

The dependent variable in this study was students’ self-reported mental health problems (L. Zhang, 2024), assessed across five dimensions: depressive symptoms, persistent sadness, chronic unhappiness, pervasive life boredom, and unresolved grief. Responses reflected experiences over the preceding seven days and were measured using a 5-point Likert scale, with response options anchored at 1 (never) and 5 (always). Higher composite scores indicated greater severity of psychological distress. The scale demonstrated strong internal consistency, with Cronbach’s alpha = 0.86.

Furthermore, we conducted factor analysis validity tests on the five scale items; the Kaiser–Meyer–Olkin (KMO) measure of sampling adequacy reached 0.86, and Bartlett’s test of sphericity yielded an approximate chi-square value of 37,212.38 with *df* = 10 at *p* < 0.001. These statistical results confirmed that the dataset was suitable for factor analysis and provided solid quantitative evidence for the construct validity of the adapted measurement instrument. Based on the eigenvalue-greater-than-one criterion combined with judgment of the scree plot inflection point, a single factor was ultimately extracted from the scale. This factor accounted for 63.96% of the total cumulative variance. Specifically, the factor loadings for all items ranged from 0.74 to 0.83, and the communalities for single items were all above 0.54, indicating satisfactory item explanatory validity for the extracted factor.

#### 2.2.3. Moderating Variables

The first moderating variable is the severity of student-reported behavioral infractions (Z. Zhang & Qi, 2024), assessed via two items adapted from the China Education Panel Survey (CEPS) student questionnaire: “I often arrive late to class” and “I often skip classes.” Responses are rated on a 4-point Likert scale (1 = never, 4 = very frequently), with higher scores indicating greater frequency of infractions. The internal consistency reliability (Cronbach’s alpha) for this two-item scale was 0.68.

The second moderating variable is migration status (Z. Zhang et al., 2026), dichotomized as “migrant status” versus “non-migrant status” based on household registration (hukou) location. Specifically, respondents whose registered residence (hukou) is located outside the county of current residence are classified as belonging to a migrant family; those whose hukou is registered within the same county are classified as non-migrant.

#### 2.2.4. Control Variables

Drawing on prior empirical literature (Eyllon et al., 2022; Hecker et al., 2016; Valencia et al., 2025), mental health problems among students are shaped by demographic, familial, and socioeconomic factors. Accordingly, this study incorporates the following control variables: gender, age, grade, ethnicity, residential location (urban vs. rural), boarding status (boarding vs. non-boarding), family economic status, and only child status. Detailed definitions and measurement approaches for each variable are provided in Table 1.

### 2.3. Data Analysis

Data analysis was conducted using SPSS 24.0 and the PROCESS macro version 4.0 (Hayes, 2017). The analytical procedure comprised three sequential steps: (1) descriptive statistics and bivariate correlation analyses were performed to examine the distributional properties and interrelationships among all study variables; (2) moderation analysis was conducted using PROCESS Model 1 with 5000 bootstrap resamples to estimate 95% bias-corrected confidence intervals for the index of the moderated effect; and (3) moderated moderation (i.e., conditional process) analysis was tested using PROCESS Model 3, also with 5000 bootstrap resamples, to derive 95% bias-corrected confidence intervals for the conditional indirect effects.

## 3. Results

### 3.1. Assessment of Common Method Bias

Common method bias was assessed using Harman’s single-factor test. Principal component analysis (without rotation) yielded nine factors with eigenvalues greater than 1, and the first unrotated factor explained only 14.44% of the total variance, well below the conventional 40% threshold. This result indicates that common method bias is unlikely to pose a substantial threat to the validity of the findings (Podsakoff et al., 2003).

### 3.2. Descriptive Statistics and Correlation Analysis

A comprehensive correlational analysis was conducted to examine the relationships among four key variables: discipline intensity, violation severity, migration status, and mental health problems. As presented in Table 2, the results indicate that discipline intensity exhibited a statistically significant positive association with both violation severity (*r* = 0.38, *p* < 0.001) and mental health problems (*r* = 0.16, *p* < 0.001), but a statistically significant negative association with migration status (*r* = −0.02, *p* < 0.01). Furthermore, violation severity was significantly negatively correlated with migration status (*r* = −0.02, *p* < 0.05) and significantly positively correlated with mental health problems (*r* = 0.12, *p* < 0.001). These results support the research Hypothesis 1.

### 3.3. Moderation Effect Analysis

**Figure 2:**
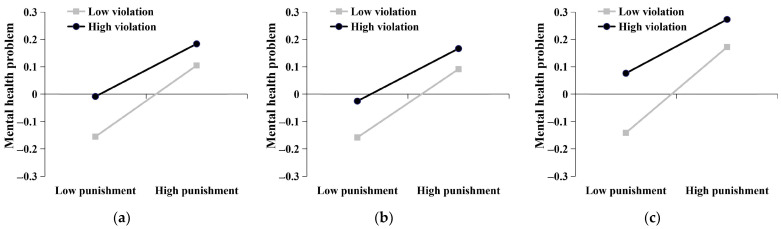
Moderating role of violation severity in the relation between discipline intensity and mental health problems for all data (**a**), non-migrant adolescents (**b**) and migrant adolescents (**c**).

Firstly, using Model 1 from the SPSS Process plugin to test the moderating effect of discipline intensity and violation severity on mental health problems. Referencing the research of Z. Zhang and Qi (2024), a stepwise modeling strategy was employed, with four moderation effect analysis models gradually incorporating different levels of covariates. Firstly, Model 1–1 served as the basic model, focusing solely on the moderating effect of discipline intensity and violation severity on mental health problems, establishing a baseline reference. Secondly, Model 1–2 included two basic demographic variables, student gender and age, to eliminate the influence of basic individual characteristics. Then, Model 1–3 further incorporated two school factors, student grade and whether they lived on campus, to eliminate the influence of school factors. Finally, Model 1–4 included other additional variables (including ethnicity, only child status, household type, family economic status, and migration status), to eliminate the influence of family and social factors and provide the most conservative effect estimation (see Table 3). Taking the most conservative and robust Model 1–4 as an example, after controlling for all additional variables, the discipline intensity could positively predict adolescents’ mental health problems (β = 0.15, *t* = 19.18, *p* < 0.001), with a confidence interval of [0.14, 0.17]. Moreover, violation severity could positively predict mental health problems (β = 0.10, *t* = 9.83, *p* < 0.001), with a confidence interval of [0.08, 0.12]. More importantly, the interaction term of discipline intensity and violation severity was significant (β = −0.04, *t* = −8.09, *p* < 0.001), with a confidence interval of [−0.05, −0.03], indicating that violation severity could regulate the relationship between discipline intensity and adolescents’ mental health issues. To more clearly illustrate the moderating effects of discipline intensity and violation severity, we used the point selection method for simple slope testing. The results of the point selection method analysis revealed that in the low severity of violations group, the intensity of discipline could significantly positively predict adolescents’ mental health problems (β = 0.17, *t* = 19.98, *p* < 0.001), while in the high severity of violations group, although the intensity of discipline could also significantly positively predict mental health problems (β = 0.13, *t* = 14.89, *p* < 0.001), the slope was significantly weaker (see Figure 2a). These results support the research Hypothesis 2.

To further examine the moderating effects of discipline intensity, violation severity, and the migration status on adolescents’ mental health problems, we conducted a moderated moderation analysis using the Process plugin’s Model 3. Similarly, we also analyzed using a hierarchical model that gradually incorporates control variables. First, Model 2–1 served as the rough model, focusing solely on the moderating effects of discipline intensity, violation severity, and the mobility status on mental health problems, establishing a baseline reference. Next, Model 2–2 included two basic demographic variables, namely student gender and age, to eliminate the influence of basic individual characteristics. Then, Model 2–3 further incorporated two school factors, namely student class and whether they lived on campus, to eliminate the influence of school factors. Finally, Model 2–4 included other additional variables (including ethnicity, only child status, household type, and family economic status), to eliminate the influence of family and social factors and provide the most conservative effect estimates (see Table 3). Taking the most conservative and robust Model 2–4 as an example, after controlling for all additional variables, the three-way interaction of the intensity of discipline, the severity of violations, and the migrant status could significantly predict the mental health problems of adolescents (β = −0.01, *t* = −2.66, *p* < 0.01). Further analysis revealed that the interaction between discipline intensity and violation severity was significant in the non-migrant adolescent group (β = −0.03, *F*(1, 17,772) = 38.70, *p* < 0.001). Specifically, the intensity of discipline could significantly positively predict adolescents’ mental health problems when the severity of violations was high (β = 0.13, *t* = 13.65, *p* < 0.001) and low (β = 0.16, *t* = 17.65, *p* < 0.001). In contrast, the interaction between the intensity of discipline and the severity of violations was also significant in the migrant adolescent group (β = −0.06, *F*(1, 17,772) = 33.70, *p* < 0.001). Specifically, the intensity of discipline could significantly positively predict adolescents’ mental health problems when the severity of violations was high (β = 0.13, *t* = 6.15, *p* < 0.001) and low (β = 0.20, *t* = 9.77, *p* < 0.001). These results support the research Hypothesis 3.

## 4. Discussion

The present study demonstrates that the intensity of punitive actions is a significant negative predictor of mental health among Chinese adolescents, thereby supporting Hypothesis 1. This finding aligns with expectancy violation theory (Burgoon et al., 2016), which posits that discipline actions exceeding normative or developmental expectations disrupt cognitive–affective equilibrium and may elicit adverse psychological responses, including heightened tension, anxiety, and depressive symptoms. These results corroborate prior empirical evidence linking punitive disciplinary practices, such as severe sanctions, exclusionary interventions (e.g., suspension), and harsh verbal reprimands, to elevated risks of depression, anxiety, and somatic complaints (Eyllon et al., 2022; Hecker et al., 2016; Valencia et al., 2025). Furthermore, qualitative insights indicate that inconsistent and excessively harsh discipline undermines adolescents’ self-worth and erodes trust within key relational contexts (e.g., teacher–student and peer relationships), thereby exacerbating psychological distress (Bell et al., 2026). Collectively, these findings suggest a dose–response relationship: higher-intensity punitive interventions by educators are associated with progressively greater severity of students’ psychological difficulties.

Furthermore, consistent with Hypothesis 2, the severity of students’ behavioral violations moderates the positive relationship between discipline intensity and mental health problems. Specifically, higher discipline intensity was associated with significantly lower levels of mental health problems in both the high-violation and low-violation groups; however, this association was significantly weaker in the high-violation group. As illustrated in Figure 2a, adolescents exhibited mental health problems below the sample mean only when teachers applied mild punitive measures in response to minor infractions; under all other conditions, namely, mild discipline for serious violations, severe discipline for minor violations, or severe discipline for serious violations, distress levels were at or significantly above the mean. These results align with just deserts theory and the legal-developmental proportionality principle: disciplinary actions should match the severity of misconduct to preserve fairness and avoid harmful psychological outcomes (Mooijman & Graham, 2018). By contrast, disproportionate discipline breeds perceptions of unfairness, triggering emotional disengagement, feelings of disrespect and interpersonal conflict. These outcomes are empirically linked to heightened psychological distress (Chen et al., 2021). Cumulative evidence further confirms that appropriate disciplinary strategies boost trust between teachers, students and families, fostering harmonious classroom interactions and sustained positive interpersonal bonds (Qi et al., 2025; Salcedo & Jimenez-Leal, 2024; L. Wang & Murnighan, 2017; Z. Zhang & Qi, 2024). Existing research yields a counter-intuitive finding: students facing harsh teacher sanctions for severe misconduct report the poorest mental health outcomes. This pattern runs counter to the proportionality principle of discipline and highlights a critical distinction between corrective discipline and retributive punishment. Teachers deliver disciplinary interventions with the goal of rectifying student misbehavior, not inflicting distress or suffering on students. Collectively, these findings suggest that mild discipline, when implemented with procedural fairness, is associated with more favorable psychological outcomes among adolescents.

Finally, this study provides empirical support for Hypothesis 3 by verifying that family immigration status significantly moderates the moderating effect of violation severity on the association between discipline intensity and adolescent mental health problems. Specifically, violation severity attenuates the negative relationship between discipline intensity and mental health problems for both migrant and non-migrant adolescents. This moderating effect is substantially stronger among migrant adolescents. This finding is consistent with the core tenets of life history theory (Stearns & Rodrigues, 2020). This framework posits that harsh, resource-poor, unpredictable early environments push people toward fast life history strategies focused on immediate survival, threat vigilance and rapid reactions, whereas slow strategies prioritize long-term investment, self-regulation and delayed gratification. Migrant adolescents endure persistent migratory stressors such as unstable housing, socioeconomic disadvantage and acculturative pressure (Ellis & Del Giudice, 2019; Han & Chen, 2020), making them prone to fast-life adaptations marked by heightened sensitivity to negative social signals, including punishment, criticism, rejection and unfavorable judgment. Prior experiments and meta-analyses have consistently verified this cognitive sensitivity pattern (Dadds & Salmon, 2003; Brance et al., 2024; Efremov, 2025; Salas-Wright et al., 2022; Sun et al., 2015). This study extends existing research by demonstrating that migrant adolescents are more responsive to disciplinary punishment than non-migrant adolescents. Only mild, well-matched disciplinary measures show no strong negative associations with mental health; all other punitive approaches correspond to worse psychological outcomes. This consistent result further proves that migrant youth are more easily affected by punishment and struggle more with social adaptation. In other words, migrant adolescents demonstrate higher psychological vulnerability when facing perceived unfair or excessive discipline. Such negative experiences further intensify their internalizing problems, such as depression and anxiety symptoms. Of course, we assume that children from migrant families experience more adverse childhood experiences; however, such experiences can also occur in non-migrant families, where they may go unnoticed, but this does not mean they do not happen. In this regard, further empirical evidence is required to demonstrate a higher prevalence of risk factors in migrant families.

It is worth noting that although all statistical associations in this study reached significance, the corresponding effect sizes were relatively small. Two primary factors jointly account for the small magnitude of the observed effects. First, the large sample of 17,788 participants encompassed highly diverse demographic profiles, age cohorts, and living environments, resulting in pronounced inter-individual heterogeneity. Compared with small, homogeneous samples, such widespread population variability inherently attenuates aggregated overall effect sizes. Second, the focal constructs assessed in this research reflect stable dispositional traits, as opposed to transient situational states. Linear correlations and predictive links between enduring trait variables are systematically weaker than those derived from short-term situational experimental manipulations, which offers a fundamental theoretical explanation for the modest effect magnitudes identified herein. Notably, these small effects carry meaningful practical implications when extrapolated to the broader target population. Even subtle correlational or predictive relationships can produce sizable cumulative population-wide impacts when generalized to tens of thousands or millions of individuals. Unlike small-scale laboratory experiments designed to elicit robust causal effects via targeted manipulations, the present population-based survey prioritizes identifying generalizable cross-population associations rather than strong context-specific effects. Accordingly, these small yet statistically reliable effects bear substantial applied value for large-scale public mental health interventions and population-level psychological governance.

## 5. Practical Implications

The findings of this study carry important theoretical and practical implications. First, to the best of our knowledge, this study is the first to systematically examine the predictive effect of teacher discipline intensity on adolescent mental health within the Chinese cultural context. Along with socioeconomic development, educational discipline and adolescent mental health have increasingly become prominent social concerns that have drawn widespread attention from parents, schools, and the general public. Based on nationally representative large-scale survey data, this study preliminarily reveals the underlying mechanisms and boundary conditions linking teacher discipline intensity to adolescent mental health problems. Consistent with common sense and existing educational perspectives, excessive teacher discipline may contribute to adolescents’ mental health. The present findings further empirically validate that discipline functions effectively only within a moderate range. Imbalanced or overly strict disciplinary practices are often associated with students’ psychological problems.

Second, this study reinforces the explanatory power of deservingness justice theory and the proportionality principle in educational settings. As fundamental guidelines for normative decision-making in daily governance and social regulation, the proportionality principle has long been applied in multiple practical domains. This study further extends its applicability to the school education context. The results indicate that mild and fair disciplinary practices benefit adolescents’ mental health, whereas excessive and overly harsh discipline significantly increases their psychological distress. Therefore, primary and secondary school teachers should strictly adhere to the proportionality principle in student management and avoid imposing extreme disciplinary actions that may hinder students’ healthy development. These findings not only help the public recognize and accept mild and fair educational discipline, but also provide empirical evidence and theoretical support for teachers to implement standardized, appropriate disciplinary behaviors.

Third, this study highlights the necessity of adopting differentiated disciplinary strategies for vulnerable student groups, particularly migrant adolescents. From the perspective of life history theory, migrant children grow up in ecologically harsh and unpredictable migration environments. Such chronic early-life adversities tend to activate fast life history developmental strategies, which are characterized by heightened sensitivity to negative social feedback, including discipline, criticism, and interpersonal rejection. This elevated vulnerability renders migrant adolescents more susceptible to depression, anxiety, and somatic symptoms. Accordingly, when handling behavioral violations of migrant students, teachers should adopt more considerate and humane disciplinary approaches centered on fairness, justice, and positive care. Targeted and inclusive discipline is often negatively associated with adverse psychological outcomes, while it has a positive relationship with the healthy development of migrant adolescents.

## 6. Limitations

Consistent with prior empirical studies, the present research has several limitations. First, the dataset adopted in this study is relatively outdated, which may fail to fully capture the contemporary impacts of educational discipline on Chinese adolescents. Notably, China’s Ministry of Education officially issued the “Rules for Educational Discipline in Primary and Secondary Schools” in 2020. This policy standardizes teachers’ legitimate, compliant, and rational use of school discipline and bears profound implications for adolescent mental health development. Therefore, future research should re-examine the association between teacher disciplinary practices and adolescent mental health problems in the post-policy implementation context.

Second, although this study draws on large-scale questionnaire data, its cross-sectional design precludes rigorous causal inference and longitudinal effect evaluation. As such, future studies may adopt longitudinal tracking designs or scenario-based experiments to clarify the causal direction and long-term developmental impacts of educational discipline on adolescent mental health.

Third, educational discipline covers a broad spectrum of practices, ranging from mild verbal criticism to severe sanctions such as school suspension and expulsion, which are characterized by substantial situational variability and gradational intensity. Likewise, student disciplinary misbehavior takes many forms. Minor classroom violations such as tardiness and unexcused absence sit alongside severe transgressions, including other types of offense directed at classmates and teachers. Restricted by the available indicators of the China Education Panel Survey, the current study only examines mild verbal disciplinary practices and low-level classroom misbehavior, and thus cannot capture the full range and complexity of disciplinary interactions and student misconduct in authentic school settings. Future research can integrate questionnaires, scenario experiments, and case studies to systematically explore how different types of disciplinary practices and student misconduct shape student mental health outcomes.

Finally, the predictor variable (teacher discipline intensity) and the primary moderator variable (violation severity) in this study were each measured with merely two items. This design led to relatively low internal consistency coefficients, and we did not conduct structural validity tests for the two constructs. In addition, this study measured adolescents’ mental health based on their emotional states over the prior week. These states may also be linked to the instability inherent in adolescence and the impact on adolescents of everything happening around them. These drawbacks partially compromise the reliability and validity of our findings. Accordingly, future investigations may adopt well-validated psychological scales with sound psychometric properties to replicate the present results and examine their generalization across large samples.

## 7. Conclusions

The present study reveals that teacher disciplinary intensity is significantly positively associated with adolescents’ mental health problems. Student violation severity further negatively moderates this association. Notably, this moderating effect is more pronounced among migrant adolescents. These findings clarify the boundary conditions of the relationship between teacher discipline and adolescent mental health.

## Figures and Tables

**Table 1:** Explanatory and descriptive statistics of control variables in data analysis.

Type	Variable Name	Items	Variable Description	M	SD
Predictor variable	Discipline intensity	Class teacher often criticizes me	1 = Strongly disagree; 2 = disagree; 3 = Agree; 4 = Strongly agree	1.51	0.65
		My parents often receive criticism about me from the teacher			
Outcome variable	Mental health problems	In the past week, you felt dispirited.	1 = Not at all; 2 = Not very; 3 = Average; 4 = Quite; 5 = Very	2.08	0.82
		In the past week, you felt depressed.			
		In the past week, you felt unhappy.			
		In the past week, you felt that life was boring.			
		In the past week, you felt sad.			
Moderator variable	Violation severity	I often arrive late	1 = Strongly disagree; 2 = disagree; 3 = Agree; 4 = Strongly agree	1.17	0.45
		I often skip classes			
	Migration status	Your current place of household registration is	1 = This County; 2 = Other County	1.17	0.38
Control variable	Individual characteristics	Student gender	0 = Female; 1 = Male	0.49	0.50
		Student age	Age at the time of the survey	14.52	1.24
	School Characteristics	Student grade	0 = grade 7; 1 = grade 9	0.48	0.50
		Boarding status	0 = Not boarding; 1 = boarding	0.32	0.47
	Family social characteristics	ethnicity	1 = Han ethnicity; 2 = Minority ethnic groups	1.08	0.28
		Only child status	1 = Only child; 2 = Not an only child	1.56	0.50
		Residential location	1 = Urban; 2 = Rural	0.54	0.50
		Family economic status	1 = Extremely difficult; 2 = Relatively difficult; 3 = Moderate; 4 = Relatively affluent; 5 = Very affluent	2.82	0.59

**Table 2:** Descriptive statistics and correlation matrix of all variables (*N* = 17,788).

Variables	M	SD	1	2	3	4
1. Discipline intensity	1.51	0.65	1.00			
2. Violation severity	1.17	0.45	0.38 ***	1.00		
3. Migration status	1.17	0.38	−0.02 **	−0.02 *	1.00	
4. Mental health problems	2.08	0.82	0.16 ***	0.12 ***	<0.01	1.00

Note: Migration status was coded as a binary variable (1 = non-migrant status and 2 = migrant status), * *p* < 0.05, ** *p* < 0.01, *** *p* < 0.001.

**Table 3:** Testing the Moderated Moderation Effect (*N* = 17,788).

Variables	Mental Health Problems (Hypothesis 2)				Mental Health Problems (Hypothesis 3)			
	Model 1–1	Model 1–2	Model 1–3	Model 1–4	Model 2–1	Model 2–2	Model 2–3	Model 2–4
Discipline intensity (A)	0.15 ***	0.16 ***	0.16 ***	0.15 ***	0.15 ***	0.16 ***	0.16 ***	0.16 ***
Violation severity (B)	0.12 ***	0.11 ***	0.11 ***	0.10 ***	0.12 ***	0.11 ***	0.11 ***	0.10 ***
Migration status (C)					0.02 *	0.02 **	0.02 **	0.03 **
A × B	−0.04 ***	−0.04 ***	−0.04 ***	−0.04 ***	−0.04 ***	−0.04 ***	−0.04 ***	−0.04 ***
A × C					0.01	0.01	0.01	0.01
B × C					0.02	0.02	0.02	0.02
A × B × C					−0.01 **	−0.01 **	−0.01 **	−0.01 **
*R* ^2^	0.03	0.05	0.05	0.05	0.03	0.05	0.05	0.06
*F*	204.14 ***	179.64 ***	128.63 ***	85.90 ***	88.80 ***	101.23 ***	83.13 ***	69.29 ***

Note: Migration status was coded as a binary variable (1 = non-migrant status and 2 = migrant status), * *p* < 0.05, ** *p* < 0.01, *** *p* < 0.001.

## Data Availability

The data presented in this study are available from the CEPS project site, subject to registration and application process. These data were derived from the following resources available in the public domain: [China Education Panel Survey] and [http://ceps.ruc.edu.cn/].

## References

[B1-behavsci-16-01209] Agnafors S., Barmark M., Sydsjö G. (2021). Mental health and academic performance: A study on selection and causation effects from childhood to early adulthood. Social Psychiatry and Psychiatric Epidemiology.

[B2-behavsci-16-01209] Bell S. L., Condon L., Nobles J., Farr M., Redwood S., Bristol’s Generation-R Young People’s Advisory Group (YPAG) Peer Researchers (Alex James, Azin Lajevardi, Elizabeth Sheldrick, Freya Milne, Madeliene Coleman and Sophie Phillips) (2026). ‘*You can’t be yourself.*’ Disciplinary behaviour management strategies and pupil mental health and wellbeing: A qualitative study of pupils’ views and experiences. Emotional and Behavioural Difficulties.

[B3-behavsci-16-01209] Black R. E., Liu L., Hartwig F. P., Villavicencio F., Rodriguez-Martinez A., Vidaletti L. P., Perin J., Black M. M., Blencowe H., You D., Hug L., Masquelier B., Cousens S., Gove A., Vaivada T., Yeung D., Behrman J., Martorell R., Osmond C., Victora C. G. (2022). Health and development from preconception to 20 years of age and human capital. The Lancet.

[B4-behavsci-16-01209] Brance K., Chatzimpyros V., Bentall R. P. (2024). Social identity, mental health and the experience of migration. British Journal of Social Psychology.

[B5-behavsci-16-01209] Burgoon J. K., Bonito J. A., Lowry P. B., Humpherys S. L., Moody G. D., Gaskin J. E., Giboney J. S. (2016). Application of expectancy violations theory to communication with and judgments about embodied agents during a decision-making task. International Journal of Human-Computer Studies.

[B6-behavsci-16-01209] Chang F., Jiang Y., Loyalka P., Chu J., Shi Y., Osborn A., Rozelle S. (2019). Parental migration, educational achievement, and mental health of junior high school students in rural China. China Economic Review.

[B7-behavsci-16-01209] Chen E., Brody G. H., Yu T., Hoffer L. C., Russak-Pribble A., Miller G. E. (2021). Disproportionate school punishment and significant life outcomes: A prospective analysis of Black youths. Psychological Science.

[B8-behavsci-16-01209] Collishaw S. (2015). Annual research review: Secular trends in child andadolescent mental health. Journal of Child Psychology and Psychiatryand Allied Disciplines.

[B9-behavsci-16-01209] Dadds M. R., Salmon K. (2003). Punishment insensitivity and parenting: Temperament and learning as interacting risks for antisocial behavior. Clinical Child and Family Psychology Review.

[B10-behavsci-16-01209] Duarte C. D., Moses C., Brown M., Kajeepeta S., Prins S. J., Scott J., Mujahid M. S. (2023). Punitive school discipline as a mechanism of structural marginalization with implications for health inequity: A systematic review of quantitative studies in the health and social sciences literature. Annals of the New York Academy of Sciences.

[B11-behavsci-16-01209] Efremov A. (2025). Psychiatry in the context of changing cultural norms: Mental disorders among migrants and refugees. Journal of Behavioral and Cognitive Therapy.

[B12-behavsci-16-01209] Ellis B. J., Del Giudice M. (2019). Developmental adaptation to stress: An evolutionary perspective. Annual Review of Psychology.

[B13-behavsci-16-01209] Eriksson K., Andersson P. A., Strimling P. (2017). When is it appropriate to reprimand a norm violation? The roles of anger, behavioral consequences, violation severity, and social distance. Judgment and Decision Making.

[B14-behavsci-16-01209] Eyllon M., Salhi C., Griffith J. L., Lincoln A. K. (2022). Exclusionary school discipline policies and mental health in a national sample of adolescents without histories of suspension or expulsion. Youth & Society.

[B15-behavsci-16-01209] Gershoff E. T. (2002). Corporal punishment by parents and associated child behaviors and experiences: A meta-analytic and theoretical review. Psychological Bulletin.

[B16-behavsci-16-01209] Hale D. R., Bevilacqua L., Viner R. M. (2015). Adolescent health and adult education and employment: A systematic review. Pediatrics.

[B17-behavsci-16-01209] Han W., Chen B. B. (2020). An evolutionary life history approach to understanding mental health. General Psychiatry.

[B18-behavsci-16-01209] Hayes A. F. (2017). Introduction to mediation, moderation, and conditional process analysis: A regression-based approach.

[B19-behavsci-16-01209] Hecker T., Hermenau K., Salmen C., Teicher M., Elbert T. (2016). Harsh discipline relates to internalizing problems and cognitive functioning: Findings from a cross-sectional study with school children in Tanzania. BMC Psychiatry.

[B20-behavsci-16-01209] Ijaz S., Nobles J., Mamluk L., Dawson S., Curran B., Pryor R., Redwood S., Savović J. (2024). Disciplinary behaviour management strategies in schools and their impact on student psychosocial outcomes: A systematic review. NIHR Open Research.

[B21-behavsci-16-01209] James P. B., Renzaho A. M., Mwanri L., Miller I., Wardle J., Gatwiri K., Lauche R. (2022). The prevalence of anxiety, depression, and post-traumatic stress disorder among African migrants: A systematic review and meta-analysis. Psychiatry Research.

[B22-behavsci-16-01209] Koenig J., Farhat L. C., Bloch M. H. (2025). From adolescence into young adulthood—The importance of a longitudinal perspective across development in child and adolescent mental health. Journal of Child Psychology and Psychiatry.

[B23-behavsci-16-01209] Krause K. H., Figueroa A., Mpofu J. J., Brener N. (2025). Report of unfair discipline at school and associations with health risk behaviors and experiences, united states, youth risk behavior survey, 2023. Journal of Adolescent Health.

[B24-behavsci-16-01209] Mooijman M., Graham J. (2018). Unjust punishment in organizations. Research in Organizational Behavior.

[B25-behavsci-16-01209] Peterson J. (2024). Observing coworkers’ violations and managers’ discipline: The effect of violation and punishment severity on coworkers. Journal of Leadership, Accountability & Ethics.

[B26-behavsci-16-01209] Piqueras J. A., Rico-Bordera P., López-Fernández F. J., Canals J., Espinosa-Fernández L., Vivas-Fernández M., PROCARE Team (2024). Subtypes of mental health difficulties and levels of resilience in Spanish adolescents. Behavioral Psychology/Psicología Conductual.

[B27-behavsci-16-01209] Podsakoff P. M., MacKenzie S. B., Lee J. Y., Podsakoff N. P. (2003). Common method biases in behavioral research: A critical review of the literature and recommended remedies. Journal of Applied Psychology.

[B28-behavsci-16-01209] Prince M., Patel V., Saxena S., Maj M., Maselko J., Phillips M. R., Rahman A. (2007). No health without mental health. The Lancet.

[B29-behavsci-16-01209] Qi C., Guo J., Liu Y., Zhang Z., Zhao G. (2025). The impact of teacher punishment intensity on parental trust in rural China: An experimental examination of a moderated mediation model. Frontiers in Psychology.

[B30-behavsci-16-01209] Qi C., Zhang Z. (2026). The impact of disciplinary intensity and number of infractions on bystander trust in teacher: The mediating role of trustworthiness. Frontiers in Psychology.

[B31-behavsci-16-01209] Qin X., Fan Y., Shen J. (2022). A study of the status quo of parental support for teacher educational disciplinary and factors influencing their support. Renmin University of China Education Journal.

[B32-behavsci-16-01209] Salas-Wright C. P., Maldonado-Molina M. M., Pérez-Gómez A., Trujillo J. M., Schwartz S. J. (2022). The Venezuelan diaspora: Migration-related experiences and mental health. Current Opinion in Psychology.

[B33-behavsci-16-01209] Salcedo J. C., Jimenez-Leal W. (2024). Severity and deservedness determine signalled trustworthiness in third party punishment. British Journal of Social Psychology.

[B34-behavsci-16-01209] Stearns S. C., Rodrigues A. M. (2020). On the use of “life history theory” in evolutionary psychology. Evolution and Human Behavior.

[B35-behavsci-16-01209] Stelmach R., Kocher E. L., Kataria I., Jackson-Morris A. M., Saxena S., Nugent R. (2022). The global return on investment from preventing and treating adolescent mental disorders and suicide: A modelling study. BMJ Global Health.

[B36-behavsci-16-01209] Sun X., Chen M., Chan K. L. (2015). A meta-analysis of the impacts of internal migration on child health outcomes in China. BMC Public Health.

[B37-behavsci-16-01209] Teodorescu K., Plonsky O., Ayal S., Barkan R. (2021). Frequency of enforcement is more important than the severity of punishment in reducing violation behaviors. Proceedings of the National Academy of Sciences of the United States of America.

[B38-behavsci-16-01209] Thomson R. M., Igelström E., Purba A. K., Shimonovich M., Thomson H., McCartney G., Reeves A., Leyland A., Pearce A., Katikireddi S. V. (2022). How do income changes impact on mental health and wellbeing for working-age adults? A systematic review and meta-analysis. The Lancet Public Health.

[B39-behavsci-16-01209] Valencia P. D., Aguilar L., Contreras-Pizarro C. H., Sequeda G., Reyes A., Gamón S., Cárcamo-Zepeda E., Piguave Holguin K. (2025). Disciplinary practices and mental health among adolescents: A person-centered approach. Current Psychology.

[B40-behavsci-16-01209] Wang L., Murnighan J. K. (2017). The dynamics of punishment and trust. Journal of Applied Psychology.

[B41-behavsci-16-01209] Wang Y., Su P., Ji J. (2021). An empirical study of parents’ attitudes towards education discipline and its influencing factors in primary and secondary schools. Contemporary Educational Science.

[B42-behavsci-16-01209] Wiggers M., Paas F. (2022). Harsh physical discipline and externalizing behaviors in children: A systematic review. International Journal of Environmental Research and Public Health.

[B43-behavsci-16-01209] World Health Organization (2024). Sexual, reproductive, maternal, newborn, child and adolescent health: Report on the 2023 policy survey.

[B44-behavsci-16-01209] Yu G. L. (2022). Chinese students’ mental health problems: The detection rate and educational implications. Tsinghua Journal of Education.

[B45-behavsci-16-01209] Zhang L. (2024). Parent-child expectation discrepancy and adolescent mental health: Evidence from “China Education Panel Survey”. Child Indicators Research.

[B46-behavsci-16-01209] Zhang Z., Li X., Juan G., Qi C. (2026). The influence of teacher care on middle school students’ social–emotional competence: Evidence from the China Education Panel Survey (2013–2014). Frontiers in Psychology.

[B47-behavsci-16-01209] Zhang Z., Qi C. (2024). Teachers’ punishment intensity and student observer trust: A moderated mediation model. Behavioral Sciences.

[B48-behavsci-16-01209] Zhang Z., Zhao Y., Huang X., Guo J., Qi C. (2025). Teacher punishment intensity and parental trust in rural China: A moderated mediation of violation severity and trustworthiness. Frontiers in Psychology.

